# Assessing the decarbonization of electricity generation in major emitting countries by 2030 and 2050: Transition to a high share renewable energy mix

**DOI:** 10.1016/j.heliyon.2024.e28770

**Published:** 2024-04-07

**Authors:** Sandra Chukwudumebi Obiora, Olusola Bamisile, Yihua Hu, Dilber Uzun Ozsahin, Humphrey Adun

**Affiliations:** a School of Management and Economics, University of Electronic Science and Technology of China, Chengdu, 611731, China; b Leeds Business School, Leeds Beckett University, Leeds, LS1 3HE, United Kingdom; c Electrical Engineering Department, College of Nuclear Science and Automation Engineering, Chengdu University of Technology, Sichuan P.R., Chengdu, 611731, China; d Electrical Engineering, Kings College London, London, SE1 8WA, United Kingdom; e Department of Medical Diagnostic Imaging, College of Health Science, University of Sharjah, Sharjah, 27272, United Arab Emirates; f Research Institute for Medical and Health Sciences, University of Sharjah, Sharjah, 27272, United Arab Emirates; g Operational Research Centre in Healthcare, Near East University, TRNC Mersin 10, Nicosia, 99138, Turkey; h Energy and Environment Division, CEPMLP, University of Dundee, Scotland, United Kingdom

**Keywords:** Decarbonization, EnergyPLAN, Energy transition, Low-carbon electricity, Renewable energy

## Abstract

The urgent need to mitigate the severe environmental impacts of climate change necessitates a transition to a low-carbon energy infrastructure, crucial for decarbonization and achieving global sustainability goals. This study investigates the decarbonization trajectories of five major economies and significant carbon emitters: the United States of America (USA), China, Japan, Germany, and India. We focus on evaluating two decarbonization scenarios for power generation. Scenario 1 explores the use of a generic storage system for reducing critical excess electricity production (CEEP), maintaining the same thermal power plant capacity as in the reference year 2021. In contrast, Scenario 2 models thermal power plants to meet the exact electricity demand without introducing a new electricity storage system. The primary aim is to assess the feasibility and implications of achieving a 100% share of renewable and nuclear energy by 2030 and 2050 in these countries. EnergyPLAN software was utilized to model and simulate the electricity systems of these countries. The two scenarios represent different degrees of renewable energy integration, demonstrating possible transitional pathways towards an environmentally friendly electricity generation system. The study provides a comparative analysis of the outcomes for each country, focusing on carbon emissions reduction and the impact on annual total costs in 2030 and 2050. Results show that by 2030, China could reduce its emissions by 88.5% and 85.14% in Scenarios 1 and 2, relative to 2021 levels. From the two scenarios considered in all the countries, India records the highest percentage reduction while Germany has the least percentage emission in reference to 2021, with a potential decrease of 90.63% and 52.42% respectively. By 2050, carbon emissions in the USA will be reduced by 83% and 79.8% using Scenario 1 and Scenario 2 decarbonization pathways. This research significantly contributes to understanding the decarbonization potential of global electricity generation. It provides vital data for policymakers, energy planners, and stakeholders involved in developing sustainable energy policies.

## 1 Introduction

During the 2015 United Nations Framework Convention on Climate Change (UNFCCC) Conference of the Parties in Paris, world leaders reached a consensus on limiting global temperature rise to below 2 °C by 2100 relative to pre-industrial levels. This consensus sparked a multitude of regional climate targets aimed at achieving this goal, primarily by reducing greenhouse gas emissions [1]. The carbon budget to stabilise global warming at 1.5 °C is becoming increasingly constrained despite global climate mitigation efforts [1]. Transitioning to a net-zero emissions by 2050 is essential to stay within the remaining carbon budget, particularly in light of rising emissions [2]. Several scenarios have been developed to show how the Paris target could be reached [[3], [4], [5]]. Most scenarios require the complete decarbonization of the power system by mid-century [6]. Around two-thirds of Greenhouse gas (GHG) emissions stem from energy production and use, therefore it is important to emphasize transitioning from fossil fuels in power generation, as well as the supply chain of energy supply [7]. To meet climate goals, progress in decarbonizing the power sector needs to accelerate further.

The International Renewable Energy Agency [8] states that the process of switching to renewable energy is a strategic direction that will change the world's energy sector from a fossil fuel-based sector, which, in turn, will drive down global emissions. An energy transition entails a profound, extensive, and long-lasting reform of the energy sector within a particular techno-institutional framework, which includes the participation of a vast array of technologies as well as organisational and institutional structures. The need to reduce energy-related carbon dioxide (CO_2_) emissions in an endeavour to mitigate or at least limit climate change is the primary impetus for this shift. Decarbonization of the energy industry requires an immediate and all-encompassing worldwide response [8]. Also, shifting from fossil fuels to clean or low-carbon technologies is a high-value policy for climate change mitigation.

Based on the Intergovernmental Panel on Climate Change (IPCC) [9] report, holding warming temperature to below 2^o^C requires significant emissions reduction by 2030, and net-zero CO_2_ emissions by 2050. Since the power sector constitutes a significant share of global emissions, focusing on cutting emissions in power generation is important. The imminent challenges have already been identified by the International Energy Agency [10]. Thus, a change to inclusive, low-carbon economies becomes imperative [11]. The necessary energy transition will be extensive, requiring a wide range of policies, financial incentives, research and development efforts, and behavioural shifts, among other things [12]. Transitions are more likely to take place gradually in energy systems because of the large capital involved and the long lifespan of fossil technologies. Energy system policies made today will have lasting effects, hence strategic energy decisions must consider medium-to long-term concerns as well as short-term needs [13]. Achieving a substantially decarbonized global economy by the middle of this century requires extensive changes to the current system, concurrent with the continued growth of the global population and economic output.

Even though a variety of strategies can aid in mitigating climate change, renewable energy is the most advantageous method for achieving the majority of necessary emission reductions at the required rate; thus, the proportion of renewable energy must increase [14]. A summary of some existing literature that reflects the state-of-the-art in this field is presented in Table 1.

**Table 1:** Summary of existing literature on power system decarbonization.

Ref	Scope/case study	Aims/Novelty	Methods/Models	Key Findings/Conclusions
Bamisile et al., 2022 [1]	China	To determine the techno-economic net-zero requirements for net zero attainment. The novelty is in aggregating government plans and the techno-economic requirements for a 100% renewable energy integration.	EnergyPLAN	With the Proposed pathway by the government, net-zero emission cannot be achieved by 2050 but the optimized strategies provide a clearer and faster decarbonization pathway.
Bamisile et al., 2022 [2]	East Africa	Proposes techno-enviro-economic solutions for East Africa's decarbonization by utilizing geothermal energy. The novelty is in the presentation of a new method towards achieving zero-carbon in enhancing the energy-water-good nexus with geothermal energy.	EnergyPLAN modelling 12 different feasible scenarios for decarbonization.	The potential for renewable energy resources particularly geothermal in resolving East Africa's energy crises is highlighted.
Handayani et al., 2023 [3]	Cambodia, Laos, Myanmmar	Contributing to a better understanding of the challenges and opportunities for renewable in least-developed nations. The use of NEMO to analyze energy storage systems.	LEAP-NEMO analysis	Electricity generation will pass the energy poverty line by 2030, 2035, and 2045. Energy storage systems are set to play a crucial role in renewable energy variability balancing.
Aszodi et al., 2023 [4]	European Union	To assess the impact of phasing out nuclear power on the electricity supply characteristic. The novelty is in the high-time resolution electricity supply models.	Energy strategies analysis using IAEA's ESST framework.	Nuclear power scenarios resulted in the lowest CO_2_ levels.
Portela et al., 2023 [5]	OECD	Provide a composite indicator for decarbonization trend tracking in the power sector and offer a benchmark. The novelty is found in the development of a new measuring approach to analyze each technology's contribution towards emissions reduction.	Four-step method combining decomposition, machine learning, visualization, and index construction.	Reveals emission reduction trend across OECD as highly heterogeneous. Highlights that better utilization of renewables is possible.
Goran et al., 2023 [6]	Europe	To analyze the decarbonization of Europe's energy system towards 2060. Main contribution is the incorporation of endogenous hydrogen demand and modelling. of energy consumption and feedstock demand.	EMPIRE model	Indicates that gas usage is significantly reduced in the power sector but is instead being replaced with coal with more expansion into renewables.
Motalebi et al., 2023 [7]	US and Canada/Northern America	Contributes to growing literature on decarbonization in Canada and the US with a clear representation of both countries in a unified scope.	OSeMOSYS energy system model	The system requires significant dispatchable, low-carbon electricity to attain emission reduction.
Hosein et al., 2023 [8]	Australia	Explores the potential for long-range renewable energy planning.	Analyzing 3 scenarios. NAU, HIG, and REE.	Slight variation in inputs can affect the timing of appropriate policies and the need for them to support desired outcomes. Careful consideration is needed.
Yin et al., 2023 [9]	China, US, EU	To present an extensive Kaya identity that explicitly expresses power decarbonization and electrification.	Logarithmic Mean Divisia Index (LMDI) model.	The combined effect of electrification and power decarbonization has an important role in carbon-mitigation in these world regions.
Haein 2023 [10]	Northeast Asia	Examines the interstate power transmissions between countries to be considered in optimal technology pathways to net-zero emissions target.	Bottom-up least-cost energy system model.	Under the carbon neutrality target, interstate power trade helps earlier carbon phase out at a lower cost.

By 2050, every country can make a big difference in how much of their overall energy use comes from renewable sources. China, for instance, could increase its share of renewable energy from 7% in 2015 to 67% by 2050. This number might rise from roughly 17% to well over 70% in the European Union (EU). In a similar vein, India and the United States may each see their share of the market increase to 63% or perhaps higher [15].

This study gives a technical, economic, and environmental analysis for transitioning to a higher share of renewable energy technologies in top emitting countries globally. The objectives assessed in this work include.

• Investigating the contribution of specific renewable energy technologies to power generation under the regions considered
• Exploring future decarbonization scenarios in the power sector of the focus countries; under different technical and cost assumptions.
• Developing a model framework that incorporates the best power mixes that would achieve the CO_2_ emissions targets in these countries.
• Informed policy suggestions towards decarbonizing the energy sectors in top emitting countries.

Considering that world nations are unified in achieving carbon neutrality by mid-century, coupled with the fact that the electricity sector contributed about 38 % of global emissions in 2022 (which is the largest share by sector), this work is timely in assessing the technical and economic feasibility of decarbonization across various constraints. While previous studies in literature especially in the integrated assessment modelling (IAM) framework have considered net-zero or hard zero emission reduction in the power sector, our modelling approach has considered two important technical constraints of practicality of decarbonizing the power sector based on its intermittence by utilizing storage mechanism, and relying on a hybrid combination of renewables and carbon fuels. This work using updated economic data on a regional basis also models the economic implications of such ambitious transitioning from fossil power generation to cleaner options. This contribution is particularly crucial for informing policy and investment decisions aimed at fostering a sustainable and economically viable energy future.

In summary, this study stands out by offering a nuanced understanding of the dynamic interplay between technological advancements, economic viability, and policy frameworks necessary for a successful energy transition. By focusing on the intermittency challenges of renewable energy and the strategic use of storage mechanisms, along with a balanced reliance on renewables and carbon fuels, we provide a pragmatic roadmap towards decarbonization that respects current technological and economic realities. This holistic approach not only enhances the literature on the pathway towards decarbonization of the power sector but also serves as a valuable resource for policymakers, energy strategists, and stakeholders who are at the forefront of designing and implementing energy transition strategies. Our work underscores the importance of adopting flexible, informed, and region-specific strategies to overcome the inherent challenges of decarbonizing the power sector, thereby contributing to the global effort to achieve carbon neutrality by the mid-century in a manner that is both technically feasible and economically sound.

This study is also novel in providing a detailed technical and economic analysis of transitioning to a higher share of renewable energy technologies in the world's top emitting countries, offering a model framework to achieve CO_2_ emission targets. It contributes significantly by not only exploring optimal renewable energy technologies under specific technical constraints but also serving as a policy guide for developing nations in settling and implementing renewable energy targets. The authors understand that there are developments regarding developments of renewable (RE) technologies in some of the countries considered, however, a more detailed coverage of the optimal RE technologies in meeting the targets upon certain technical constraints is worth investigating. Additionally, this work can serve as a policy guide to developing nations in building RE targets and implementations.

## 2 Energy situation of countries considered in this study

### 2.1 The United States of America (USA)

**Fig. 1:**
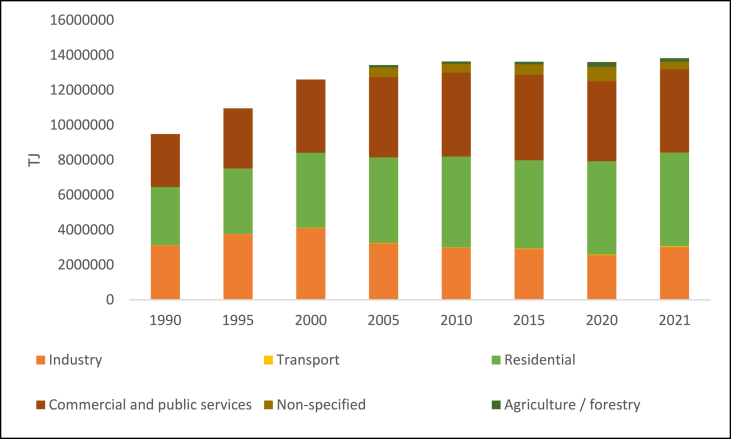
US Electricity consumption by sector, 1990–2021.

Source: EIA, 2022.

Electricity is produced in the United States using a wide range of energy sources and technologies. The three main types of energy used to generate electricity are nuclear energy, renewable energy sources, and fossil fuels (coal, natural gas, and petroleum) [16]. The majority of the world's electricity is produced by steam turbines that use various forms of energy, including fossil fuels, nuclear power, biomass, geothermal, and solar thermal. In the United States, gas turbines, hydro turbines, wind turbines, and solar photovoltaics are also integral components of the nation's energy infrastructure. Fig. 1 shows the consumption by sector in the USA, which shows that buildings and industries are large consumers of electricity.

**Fig. 2:**
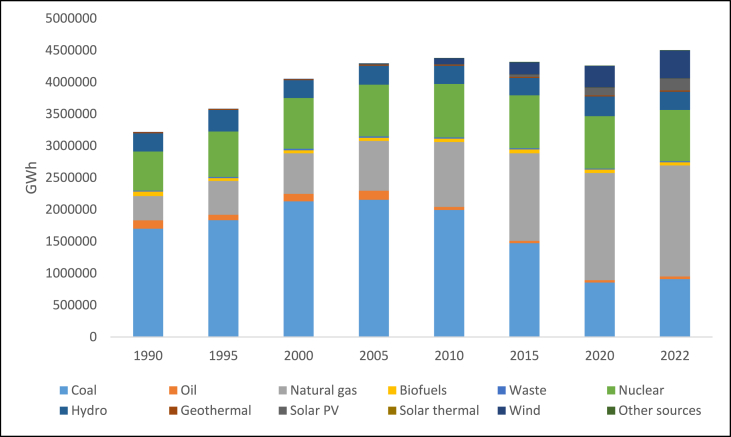
U.S. electricity generation by major source, 1990–2022 [17].

From Fig. 2, a trend of increasing fossil fuel consumption for electricity generation is observed, with the largest share of 22% being from coal in 2021. Nuclear energy contributed 19% to electricity generation in 2021. Despite the reliance on fossil fuels, renewable energy has also been on an upward trend, with wind energy contributing significantly (Fig. 2)

The United States generates power from a wide variety of renewable energy sources; in 2021, these sources accounted for around 21.5% of the country's total electrical generation, with hydroelectric plants accounting for nearly 6.3% of the total. In 2021, wind energy accounted for 9.2% of all U.S. electricity generation and 46% of all electricity from renewable sources. In the same year, biomass accounted for approximately 1.3% of the nation's total power generation and 6.7% of renewable energy production. In 2021, solar energy contributed approximately 2.8% of the total electricity produced in the United States and 13.5% of the renewable electricity. In the same year, geothermal power facilities generated nearly 0.4% of the nation's total electricity and approximately 2.0% of the electricity from renewable sources.

### 2.2 China

**Fig. 3:**
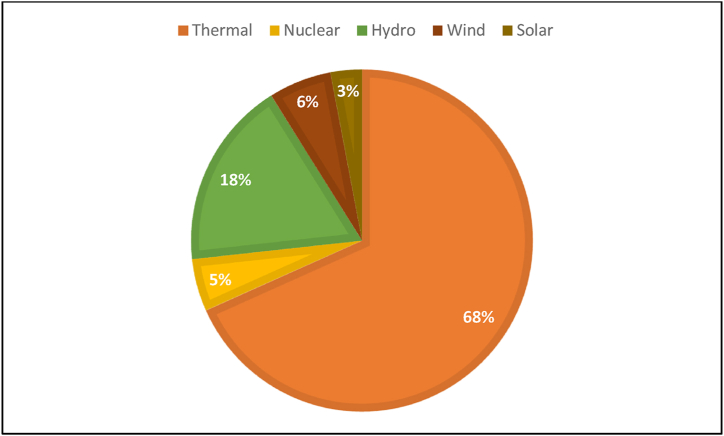
Power generation mix in China, 2019 [21].

Since 2011, China has produced the most power globally, and this trend is expected to continue. With 1777.08 GW installed capacity at the end of 2022, China holds the top spot globally for installed power-producing capacity [18]. Non-fossil energy sources produced 30.3% of the electricity in 2017, up 10.9% from 2016. Fig. 3 shows that coal, which made up 69% of the power-generating mix in 2019, is the primary source of electricity in China [19]. This contributes significantly to China's GHG. However, the amount of power produced from renewable sources has been continuously rising, from 615,005 GWh (17.66% of the total) in 2008 to 2,082,800 GWh (27.32% of the total) in 2020 [20].

China, which has numerous rivers and steep mountain valleys, has greatly benefited from the expansion of its water resources in recent years. Approximately 17% of the world's current electric power capacity comes from hydropower. After an additional 4.2 GW of capacity was installed, the total hydropower capacity reached 356 GW in 2019, and the expected hydropower output reached 1302 TWh [22]. The greatest market for solar thermal energy and photovoltaics is China. In 2020, China produced 7623 TWh of electricity; 261.1 TWh of that amount, or 3.43%, was produced using solar energy [23]. As of 2022, the percentage of electricity generated from non-fossil fuels moved up to 37% with the largest source being hydro at 14%, wind at 9%, and nuclear and solar at 5% and 5% respectively. Coal as an energy source dropped to 63% showing a gradual decline in reliance on fossil energy in the country. As of November 2022, the proposed capacity of China's nuclear power plants stood at around 178 GW [24].

### 2.3 Japan

**Fig. 4:**
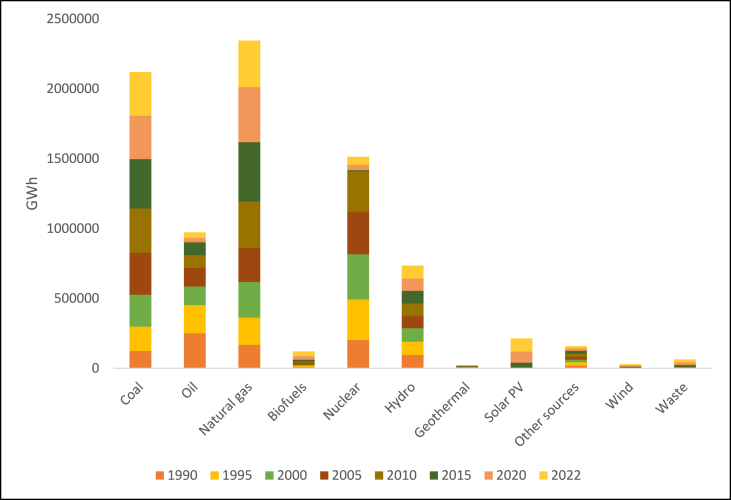
Japan's electricity generation by major source, 1900–2020 [17].

According to the IEA, Japan's 88% of the total power output in 2021 was derived from fossil fuels (Fig. 4). Japan consumes about 96% of its energy resources from importation, which makes it the fifth-highest country in terms of carbon intensity. Japan, however, has greatly diversified its energy mix in recent years. By putting a strong emphasis on renewable energy, it has worked to overhaul its energy systems and policies. The outcomes thus far are pleasing, but there is still much space for development in the nation [17].

During the early phases of Japan's industrialization period, hard coal or lignite was extremely important. However, this principal energy carrier was not frequently exploited for energy production. Only 5% of the energy required in the 1980s was met by coal. After 2011, the percentage climbed a little, and in 2014, it was close to one-third of the total power generated. Because the massive units cannot react fast to changes and require hours to heat up and cool down, coal power plants are mostly utilized for base load and are avoided if feasible.

Similar significance is attached to oil in Japan's industrialization process. However, over time, it produced a lot more electricity. Mineral oil was used to produce 46% of the nation's power in 1980; this percentage continuously decreased until 2010. To make up for the shutdown of nuclear reactors, strategic reserves were revived in 2011, and oil contributed 18% of the nation's energy production in 2012 before falling to 11% in 2014 [25]. The Japanese industry continues to be dependent on mineral oil. Oil is still a significant source of energy, although not as much as it once was. When necessary, strategic reserves (power plants) can be activated to meet spikes in demand or make up for the failure of other power facilities.

Since the 1980s, natural gas, or LNG, has gradually grown in importance for the Japanese energy industry. Although this percentage of the overall energy output in 1980 was only 15%, LNG became more significant during the following decades, notably in 2011, when it generated 40% of the entire power demand [26].

Japan has been a commercial user of nuclear energy since 1966. Nuclear energy became a national strategic priority in 1973 as a direct response to the oil shocks of the 1970s (oil imported from the Middle East powered around 66% of the nation's electricity in 1966) [27]. With the aid of contemporary technology, Japan's already pronounced high dependence on fossil fuel imports should be reduced while simultaneously supplying enough power to maintain the nation's economic expansion [28]. At the beginning of 2011, 30% of Japan's energy consumption was met by 56 reactors with a combined installed capacity of 47.5 GWe (net). By 2017 and around 50% by 2030, nuclear energy was projected to provide 41% of Japan's power to achieve the country's emissions reduction targets. Due to the Great East Japan Earthquake and the ensuing nuclear accident at the power plant Fukushima Daiichi, these plans were abruptly abandoned. Due to the badly damaged and rendered inoperable reactors at Fukushima, the installed capacity was reduced to 44.6 GWe (net). Additionally, all other reactors were turned off and examined. At the moment, 24 of the 43 operating units that might be restarted are awaiting restart permission. The only nuclear reactors in Japan that are now producing energy are the two reactors of the Sendai nuclear power station in Kagoshima, which were reactivated in August and October 2015; the other reactors are presently undergoing maintenance [28].

### 2.4 Germany

In 2013, about 45% share of power generation sources was from coal. This reduced to 24% by 2020, of Germany's power was generated from coal. Despite the reduction in reliance on coal, it remained Germany's principal source of power in the first half of 2021 [29]. Coal-based electricity output decreased by 51% (−138 TWh) between 2015 and 2020. Germany still depends on fossil fuels despite the fall in coal use. Since 2015, fossil gas has grown its percentage by 67 per cent and now accounts for 16 per cent of Germany's power mix. While overall fossil generation has decreased by just 31% since 2015, coal generation has decreased by 51% since that time [30].

In 2021, gas-fired power plants provided roughly 51 TWh of electricity, which was less than the level of 57 TWh in 2020. In 2021, hydropower generated 19.4 TWh of electricity, up from 18.2 TWh in 2020. Biomass generated slightly more power in 2021 (43 TWh) than in 2020 with almost no change in installed capacity [27]. Between 2015 and 2020, nuclear energy output dropped from 92 TWh to 64 TWh (a 30% decline). Even though the installed capacity is presently 10 GW, producing 11% of Germany's power, legislation enacted in 2011 requires all existing nuclear facilities to shut down by the year 2022 (Brown, 2021a). Gerhard Schroeder's coalition government decided in 2002 to completely phase out nuclear power by 2022 [31]. Beginning in 2007, the issue attracted increased interest because of the political ramifications of the energy conflict between Russia and Belarus, and again in 2011 following the Fukushima I nuclear catastrophe in Japan [8].

**Fig. 5:**
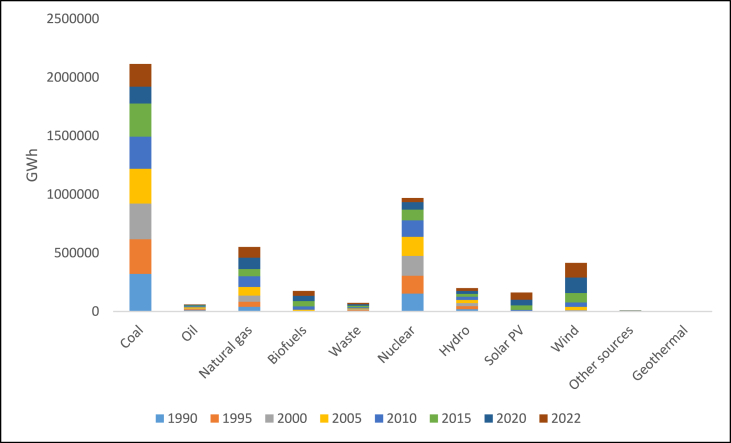
Installed net power generation capacity in Germany, 2002–2021 [17].

Since 2015, renewable energy production has doubled, making up about 45% of Germany's power generation (Fig. 5). On the German public grid, renewable energy had a share of 50.9% in 2020. Solar energy accounted for 10.5% of total generation, and wind power for 27 %. Hydropower made up 3.8 %, while biomass made up 9.7% [32]. As a consequence, renewable energy sources first surpassed fossil fuels in 2020, setting a historic precedent. The 2020 generation from fossil fuel rate was 44%. The increase in the share of wind and solar by 14%, caused coal to reduce by 19 % [30].

### 2.5 India

**Fig. 6:**
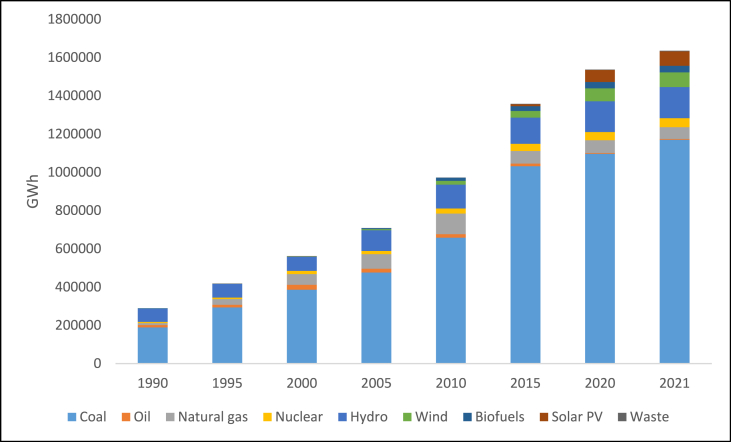
Installed net power generation capacity in India, 2022 [17].

With a total installed power output of 403.759 GW as of 2022, India is the world's second-largest consumer of energy and the third-largest producer of energy [33]. A significant contributor to India's electricity generation is coal. According to Fig. 6, coal accounts for around 50.7% of India's total installed capacity. India generated 9% more power from coal between 2015 and 2020. Although the proportion of renewable energy in India's power mix has grown over the past five years, its growth in absolute terms has lagged behind the country's expansion in electricity consumption. Despite its power market share declining from 77% in 2015 to 71% in 2020, this increased coal-based power output by 9% (+75 TWh). Additionally, India added 45.2 GW of additional coal capacity over these five years, surpassing Germany's total installed coal-based power capacity of 43 GW [23].

In terms of the overall generating mix, nuclear power has been essentially consistent, but its share is still rather small when compared to other sources. Nearly 6.8 GW capacity of plants are operational at the moment, while 5.4 GW capacity is being built. Although the World Nuclear Association (2019) predicted that between proposed and planned projects, 80 GW more nuclear power may be placed in the long run, other assessments are more conservative on the amount of realistic capacity that might be delivered.

Due to the delayed development of new plants and a decline in capacity factors, hydro has lost market share in the overall capacity and generation mix. The potential for additional expansion of hydroelectric plants is enormous, according to CEA [34]. Even though 11 GW of run-of-river and reservoir plants were still under construction as of March 2017, there were already up to 40 GW of these facilities, according to CEA, and there is still room for 95 GW more. By the conclusion of the 2016–17 fiscal year, 5 GW of pumped hydro storage facilities were in operation, 1 GW were under construction, and the CEA estimates 97 GW in further pumped storage potential at 63 distinct sites.

India expanded both its renewable and fossil fuel generation as a result of the country's rising energy consumption. Over the previous five years, India's power demand climbed by 18% (+205 TWh). The amount of electricity produced also rose by 18% (+202 TWh), with fossil generation accounting for 43% of this increase, which climbed from 913 TWh in 2015 to 999 TWh in 2020. The percentage of renewable generation increased from 16% (189 TWh) to 22% (298 TWh) over the same period, replacing coal with 6% of the power market [23]. India had 152.36 GW of installed renewable energy capacity as of January 2022, or 38.56% of all installed power capacity [27].

## 3 Materials and methods

### 3.1 ENERGYPLAN simulator

The model's emphasis is on integrating future technology and energy systems. As a result, the model incorporates thorough modelling of many technologies, including compressed air energy storage (CAES), biomass gasification, renewable energy technologies, electric vehicles, and electrolysers.

In contrast to stochastic models or models that employ Monte Carlo techniques, Energy PLAN is a deterministic model, which means that given the same input, it always produces the same outcomes. Demands for electricity, heating, and fuel, energy technologies, economic variables in various industries are general inputs. Some outputs from the model include hourly or yearly electricity generation, emissions, excess electricity, and economic variables.

Energy balances and yearly productions, fuel consumption, import and export of power, CO_2_ emissions, and total annual system expenditures are examples of outputs. The model is separated in this fashion into the four primary input tab sheets for Demand, Supply, Balancing and Storage, and Cost, as well as an extra tab sheet for Output where the user may select from many ways to display output components. Sub-tabs are further separated into each tab, often adhering to the logic of various energy sectors [35]. Since the major goal of EnergyPLAN is to discover and analyze individual energy technologies and uncover different investment possibilities rather than using a macroeconomic method to evaluate various energy policies, this characterizes EnergyPLAN as a bottom-up tool. The short-term electricity consumer costs and short-term district heating expenses are the main goals of the market economic simulation, which is based on a short-term marginal price market model. This approach optimizes only the supply side of the energy system and only employs variable costs [35].

### 3.2 Data collection

**Fig. 7:**
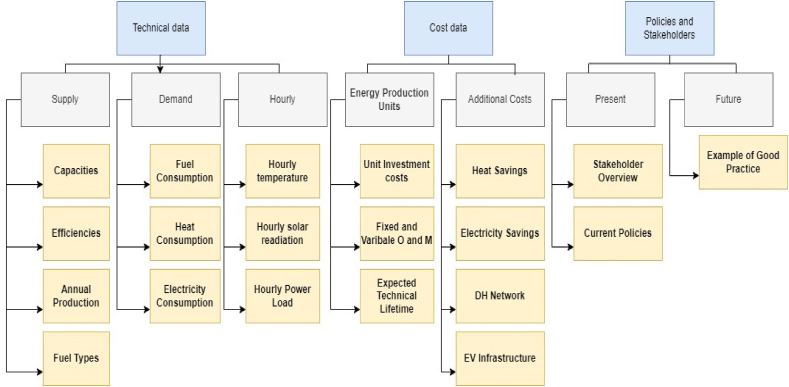
Overview of types of data and information used for analysis [36].

Data and information are gathered for two major reasons: to feed the EnergyPLAN model so that energy system assessments can be performed and to gain knowledge of institutional and policy frameworks so that policy recommendations can be made. Fig. 7 provides a structured overview of the major categories of data. While the other data and information are utilized for policy and stakeholder analysis, the first two components, technical and economic data, are used for studies of the energy system. The economic data utilized for this study is presented in the supplementary document (Table S1 to S8).

For uniformity and comparison, the electric efficiency of all the thermal power plants in each country is taken as 0.45 (45%) in all cases. The generation capacity and the correction factor are also the input data used in modelling renewable energy technologies. The renewable energy resource distribution profile is also essential in calculating the annual power production and the capacity factor for each of the systems. The capacity factor for each renewable energy system in each country is calculated using the equation:

(1) $$capacity\;factor=\frac{Electricity\;generared\;in\;a\;year\;from\;the\;renewable\;source}{Electricity\;capacity\;of\;the\;renewable\;source\times 8760}$$

To replicate the hourly, daily, and seasonal variations in energy demand, as well as the intermittent hourly behaviour of energy-producing units, hourly distribution data is required. Data on solar radiation is also obtained from Meteonorm, and the EnergyPRO software's methodology is used to determine solar output. The technique considers the location's geographic parameters, the panel's orientation (inclination of the surface, orientation of the inclined plane), and the technical specifications of the solar panel, including efficiency coefficients, angle factors, and dimensions. Finally, CSP and run-of-river hydro distribution curves may be acquired from the most recent version of ENERGYPLAN s distribution library.

### 3.3 Simulation methodology

The simulations will be performed for two decarbonization scenarios and two future years (2030 and 2050). The electricity demand values for these two years are predicted using the average percentage change over the last 30 years to calculate the demand for the next 30 years. The decarbonization scenarios will be on the assumption that all the electricity demand in 2030 and 2050 in each of the countries is supplied by 100% RES and nuclear energy. The modelling scenarios were developed to highlight certain challenges that could be encountered in transitioning to a 100% (or near 100%) renewable energy-powered electricity sector. Recent technological developments have shown that battery storage is becoming cheaper. The encouraging trend in the decreasing costs of battery storage is a pivotal factor in addressing one of the critical challenges associated with renewable energy integration – intermittency. As renewable sources such as solar and wind energy are inherently variable, efficient and cost-effective energy storage solutions play a crucial role in maintaining grid stability and ensuring a reliable power supply. This is the basis upon which the first scenario is modelled to reflect a dynamic assessment of the potential impact of storage penetration on mitigating the challenges posed by intermittency in a transition to a 100% (or near 100%) renewable energy-powered electricity sector. We have also modelled a scenario to reflect a possible future where despite the penetration of renewable energy technologies, fossil fuel power plants remain a significant part of the energy landscape. This scenario serves as an exploration of the complexities and uncertainties surrounding the transition to renewable energy. In modelling this second scenario, we acknowledge the intricate balance between renewable energy adoption and the persistence of fossil fuel power plants. Despite advancements in renewable technologies, certain practical and economic considerations may lead to the coexistence of traditional energy sources with their greener counterparts.

Each of the two decarbonization modelling frameworks is described.

• In the first scenario, the critical excess electricity production (CEEP) will be covered by a generic storage system. In this scenario, there is no change in the thermal power plant capacity which will be the same as the value of the reference year (2021).
• In the second scenario, there will not be a new electricity storage system. However, the thermal power plant capacity value will be modelled to the exact amount to meet the required demand for electricity using the thermal power plants.

**Fig. 8:**
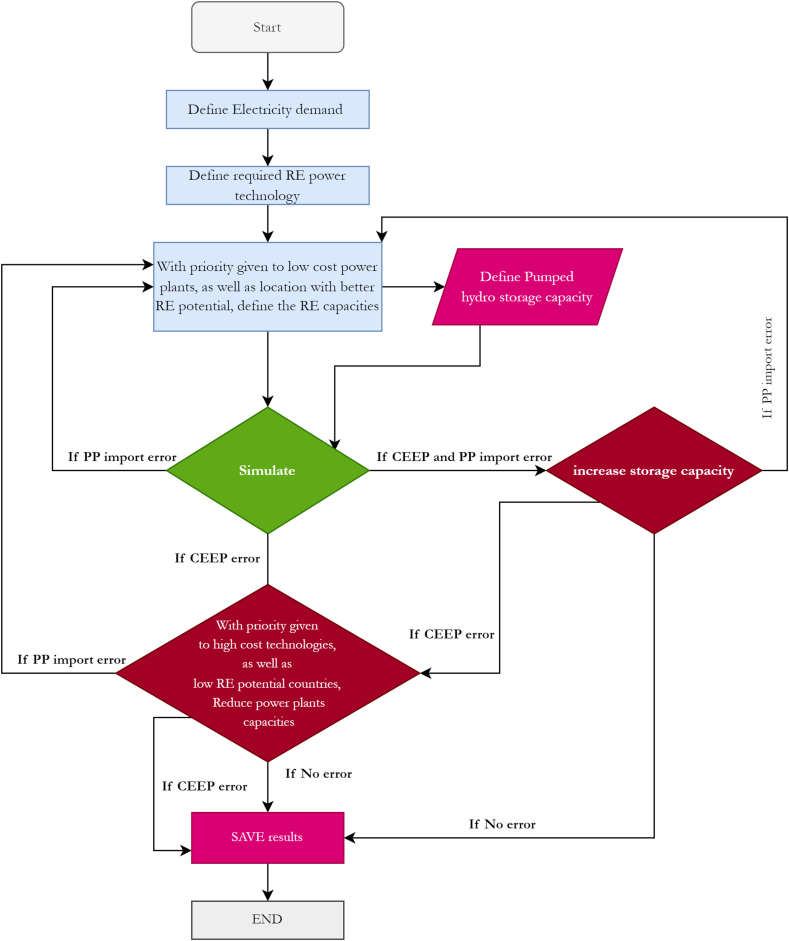
Methodology schematic.

**Fig. 9:**
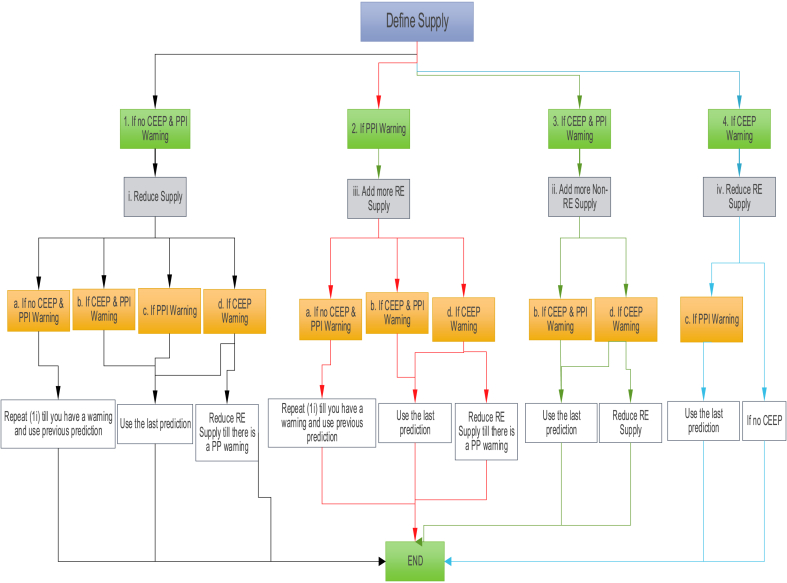
Optimization simulation flowchart.

The methodological step is shown in Fig. 8. The validation of the model is first estimated as reflected in the results in section 4.1. This represents the baseline scenario, where historical data like the electricity demands, and the thermal power plant capacity are inputted into the modelling tool. The output is compared with the retrieved data from the literature to assess the validity of the model (See section 4.1). In the modelling of the scenarios, the input data of future electricity demand is estimated and used as input. In the 100% scenarios, an optimization process (Fig. 9) is implemented in the modelling environment to estimate the optimal renewable energy capacity to meet future demands, contingent on the critical excess electricity production (CEEP) and power plant input (PPI). A CEEP warning shows that surplus electricity production persists once annual demands are fulfilled. To address this, the capacity of renewable energy supply is systematically reduced until reaching the optimal technical scenario. A PPI warning shows that the power plant output falls short of satisfying annual demand. Enhancing the capacity of power plants, prioritizing renewable energy technologies, will continue until reaching the optimal capacity.

The input data for the five countries under consideration is obtained from various sources like online websites, research articles, and publications. The input data utilized in this study cover the relevant points needed in this study. The data gathered were for a reference year (2021) and the two future years (2030 & 2050). Some relevant input data are presented in Table 2 to Table 3. Table 2 shows the electricity demand from the 5 countries in 2021. Also, the table shows the value of the capacity of fossil fuel thermal power plants in each of the 5 countries. It also shows the projected electricity demand in 2030 and 2050 for all 5 countries. Table 3 shows the carbon price fixed to curtail carbon emissions in all 5 countries.

**Table 2:** Electricity Demand and fossil power plant capacity in 2021 and input electricity capacity in 2030 and 2050 [23,29,34,37].

Country	Electricity demand (TWh/year)	Fossil fuel thermal power generation Capacity (GW)	Electricity demand in 2030 (TWh/year)	Electricity demand in 2050 (TWh/year)
USA	4189.28	840. 2	4284	5138
China	8466.32	1296.8	10300	13600
Japan	958.53	312.8	952.65	939.72
Germany	562.41	7.5	658	960
India	1365.43	235	2341	5921

**Table 3:** Carbon price in all the five countries (World Bank, 2021)

Country	Carbon Price (US$/tCO_2_e)
USA	31
China	9
Japan	2
Germany	33
India	10

## 4 Results and discussion

In this section, the results are obtained from the simulation performed for the decarbonization scenarios for each country in 2030 and 2050. Also, the discussion of the results as they relate to the set objectives of the study is presented in the section.

### 4.1 Reference year (2021) results/model validation

A constructed model must be validated to confirm its precision and applicability. The main goal of model validation is to estimate how well the model is likely to perform on future data. In this study, the model validation is performed using input data from a reference year (2021) and comparing the results from the simulation to the values supplied by each of the countries. The results used in the comparison are the electricity sector CO_2_ emission levels (Mtons/yr) and the renewable share of total electricity production values (%). Table 4 shows the comparison used for the model validation.

**Table 4:** Simulation results for 2021 used for system validation

Country	CO_2_ emissions		
	Value (Mtons/yr)	Simulation (Mtons/yr)	Percentage difference (%)
USA	1576.98	1597.54	1.3
China	4615.31	4664.82	1.1
Japan	459.14	446.34	2.9
Germany	205.82	203.73	1.0
India	865.91	862.21	0.4

**Fig. 10:**
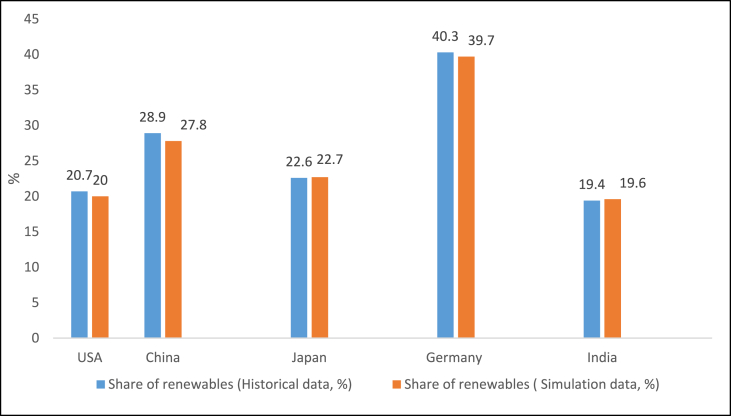
Share of renewable energy from historical and simulation data (2021).

Table 4 and Fig. 10 shows that the results from the simulation are in good agreement with the data reported by the countries. The biggest deviation in the share of renewables is 3.8% in China while the biggest deviation in CO_2_ emission is 2.9% in Japan.

### 4.2 Hourly electricity profiles for all five countries

**Fig. 11:**
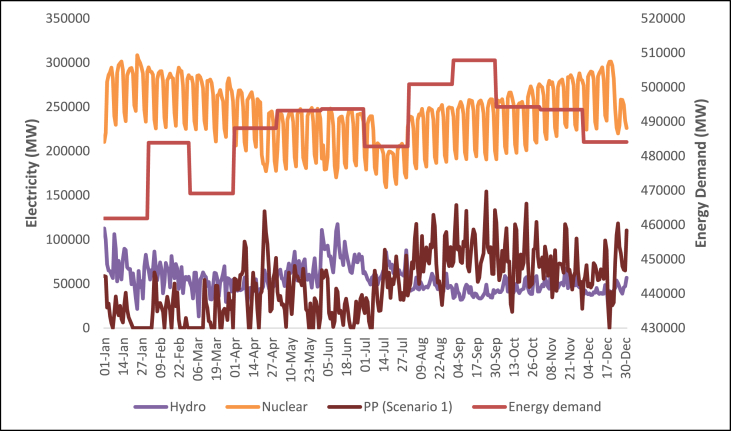
Daily electricity profile for energy demand, nuclear energy, and hydroelectricity in 2030 for the USA.

**Fig. 12:**
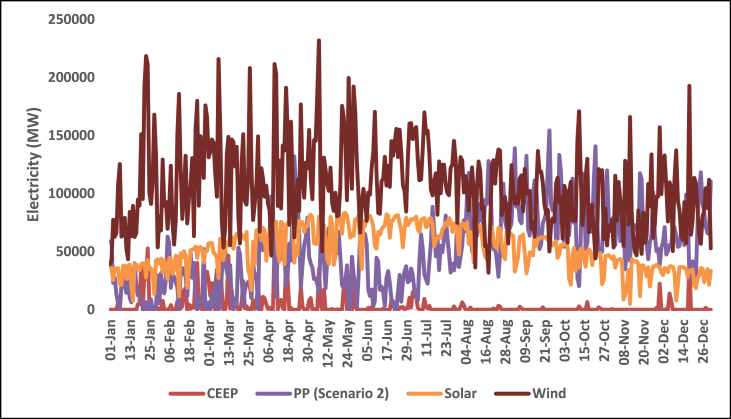
Daily electricity profile for wind energy, solar energy, CEEP, and fossil fuel power plants in 2030 for the USA.

**Fig. 13:**
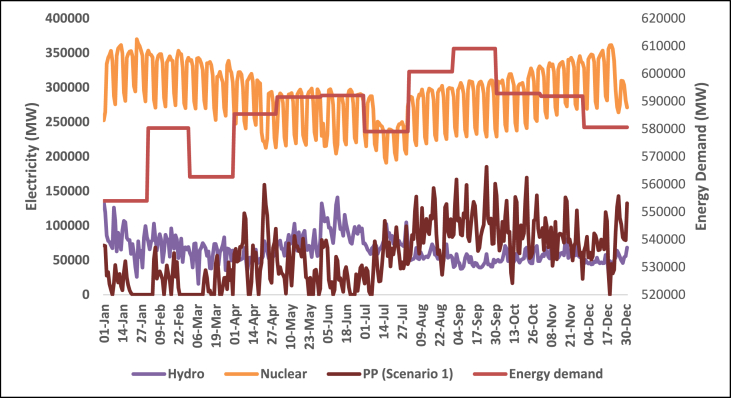
Daily electricity profile for energy demand, nuclear energy, and hydroelectricity in 2050 for the USA.

**Fig. 14:**
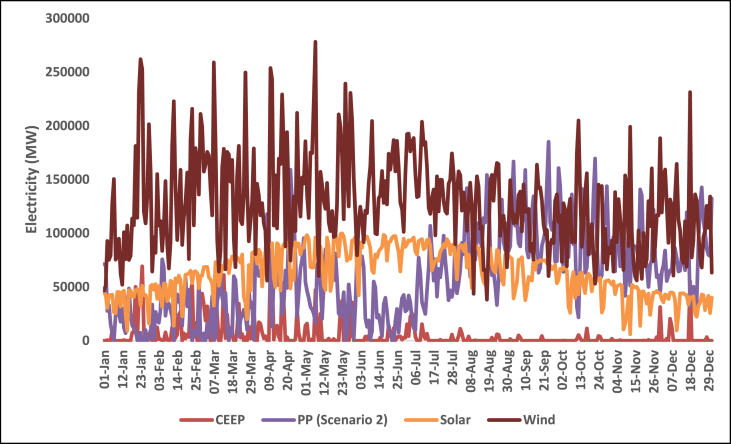
Daily electricity profile for wind energy, solar energy, CEEP, and fossil fuel power plants in 2050 for the USA.

This section shows the results of the hourly electricity profiles for a year for each country in 2030 and 2050. The profiles consist of the electricity demand together with electricity supply from renewable technologies. Also included are the hourly profiles of the electricity storage system (scenario 1) and the critical excess electricity production (scenario 2). Fig. 11, Fig. 12, Fig. 13, Fig. 14 show the hourly energy supply for the USA. Figs. S1–S16 in the supplementary document show the electricity supply for all four other countries in 2030 and 2050 respectively.

The demand for all 5 countries is shown to be steady on an daily basis all year round, the demand during the summer months is slightly higher considering the increased demand for space cooling and refrigeration. There are also variations in the hourly demand in a day which is due to various factors such as industrial activities, residential consumption, and commercial operations, which differ between countries and over time. China exhibits the highest demand for electricity both in 2030 and 2050 based on the predicted electricity generation values shown in Table 2. The somewhat steady demand for electricity in the countries means that there is a need to also ramp up the capacity of electricity generation systems that can supply constant power without fluctuations. These are known as base load power generation systems.

The electricity supply for the USA is shown in Fig. 11. Nuclear power plants provide a consistent and reliable base load of electricity, operating continuously throughout the year. The hourly profile for nuclear energy indicates a stable output with minimal fluctuations. This characteristic makes nuclear energy a reliable and predictable source of electricity, contributing to grid stability and meeting the base load requirements. Nuclear energy is shown to provide a big part of the electricity generated to cover the electricity demand in most countries.

Hydroelectricity also covers a large chunk of the electricity demand. The daily supply from hydroelectricity however is not steady. Hydroelectric power generation is subject to the availability of water resources and seasonal variations. Consequently, the hourly profile for hydroelectricity exhibits fluctuations that align with the availability of water flow. During periods of high-water availability, hydroelectric plants can generate significant amounts of electricity, while during drier seasons, the output may decrease. The hourly electricity profile for wind and solar energy exhibits their intermittent nature. Wind power generation is influenced by weather conditions, particularly wind speed and direction while solar power generation is influenced by factors such as sunlight intensity, cloud cover, and the angle of incidence. Consequently, the hourly profile for wind energy exhibits fluctuations throughout the day and across seasons. There are periods of high wind activity leading to increased electricity production, while lulls in wind speed can result in decreased generation. Similarly, the hourly electricity profile for solar energy highlights its intermittency. The high supply of solar energy happens around the spring and summer while the supply is shown to reduce towards the end of the year. Typically, the hourly profile for solar energy exhibits a diurnal pattern, with peak generation occurring during the daylight hours and minimal or no generation during nighttime. However, the availability of solar energy during the day aligns with the electricity demand profile, making solar power an important contributor to meeting daytime energy needs.

The Daily profile for fossil fuel power plants reflects their role as dispatchable sources of electricity. Fossil fuel power plants, such as natural gas or coal-fired plants, can be ramped up or down to respond to changes in electricity demand or to compensate for fluctuations in renewable energy generation. As a result, the daily profile for fossil fuel power plants exhibits flexibility and can be adjusted to meet the varying electricity demand throughout the day. The occurrence of critical excess electricity production is an important consideration for grid management. During periods of high renewable energy generation, such as when wind and solar resources are abundant, electricity production may exceed the immediate demand. This excess electricity can pose challenges to the grid's stability and may require curtailment or storage solutions to avoid disruptions. The identification of critical excess electricity production in the hourly profile is crucial for effective grid balancing and optimal utilization of renewable energy resources.

The occurrence of CEEP in all five countries also matches the daily profile for solar energy

which makes it pertinent for large-scale energy storage. This present study does not specify a particular storage device to be used to cover the CEEP, however, examples of large-scale storage devices to be used are CAES, batteries, and hydrogen. Also, the storage capacity of the pumped hydroelectric storage system, already considered in the simulation for each country, can be increased. A demand-side management plan will combine some or all of this storage to ensure that the appropriate storage systems will be available depending on the status of demand. For example, battery systems can be scalable which will make them suitable for storage and dispatch in periods when electricity demand is not large.

### 4.3 Storage and critical excess electricity production (CEEP)

This section provides the results for the CEEP and the required storage system size to cover this CEEP. The simulation methodology explained that scenario 1 in this study examines the situation whereby all the CEEP produced by each country is covered by storage systems while scenario 2 assumes that no storage system is used to cover the CEEP and the excess production can be used for various other purposes such as electricity export, electric vehicles, hydrogen production, etc.

**Fig. 15:**
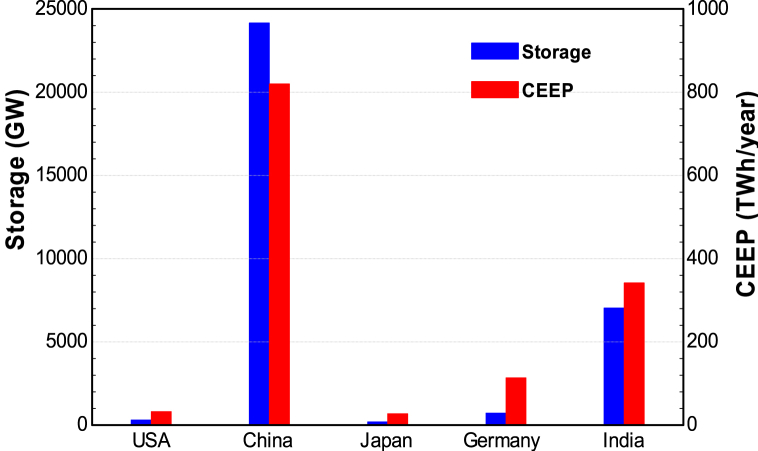
Required storage size (Scenario 1) and CEEP (Scenario 2) in 2030.

The results in Fig. 15, for 2030, show that China will require the highest storage capacity with a value of about 24190 GW. This is to cover 1110,341.7 TWh/year of excess electricity. In comparison, India requires 7040 GW of storage system to cover 341.7 TWh/year of excess electricity.

**Fig. 16:**
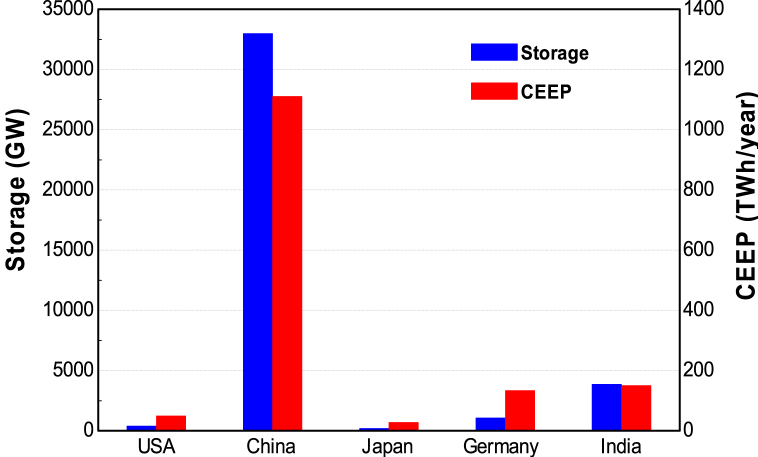
Required storage size (Scenario 1) and CEEP (Scenario 2) in 2050.

The results in Fig. 16 show a similar trend in CEEP and storage system requirements. However, while the values increase in the case of USA and China, in reference to 2030, the values are shown to decrease for Japan, Germany, and India. Japan's CEEP slightly drops from 27.18 TWh/year to 26.99 TWh/year. This is due to the projected drop in electricity demand in Japan between 2030 and 2050.

### 4.4 Emissions reduction

**Fig. 17:**
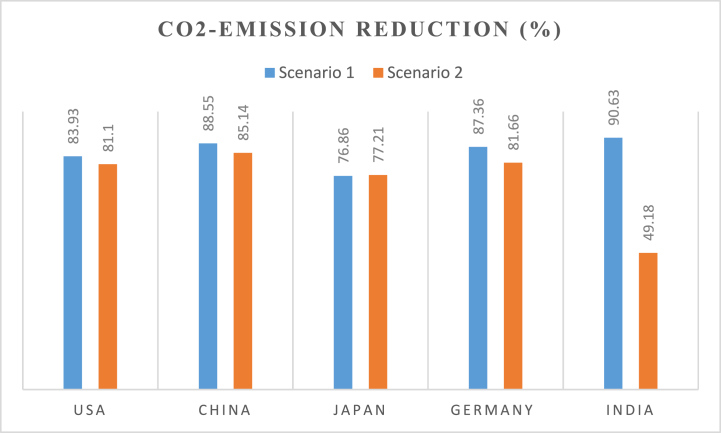
CO_2_ emission reduction in both scenarios in 2030.

**Fig. 18:**
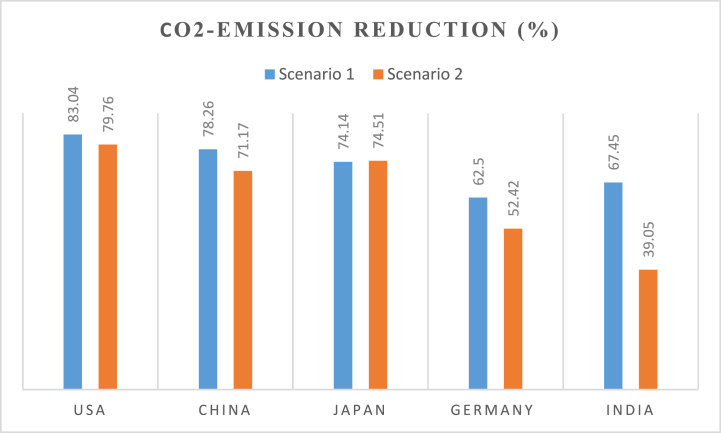
CO_2_ emission reduction in both scenarios in 2050.

Fig. 17, Fig. 18 show the CO_2_ emissions reduction that can be achieved from both scenarios for 2030 and 2050. The percentage reduction reported is in comparison to the CO_2_ emissions values in the reference year (2021). Fig. 17 shows that 83.93% of the CO_2_ emissions in 2021 can be eliminated by 2030 in scenario 1 while scenario 2 can reduce the emissions by 81.1%. Similar results are observed for the other four countries. China's emissions can reduce by 88.5% in Scenario 1 and 85.14% in Scenario 2.

The highest percentage reduction is in India, which can reduce its emissions by 90.63% in scenario 1. However, its emissions reduction in scenario 2 is 49.18%. This is due to India's need to increase generation from fossil fuel power plants to cover some of its base load demands in 2030. The results for 2050 (shown in Fig. 18) exhibit the same trend. However, the percentage reduction values are lower compared to the 2030 values. This is due to the increase in electricity demand for 2050.

### 4.5 Total annual costs

**Fig. 19:**
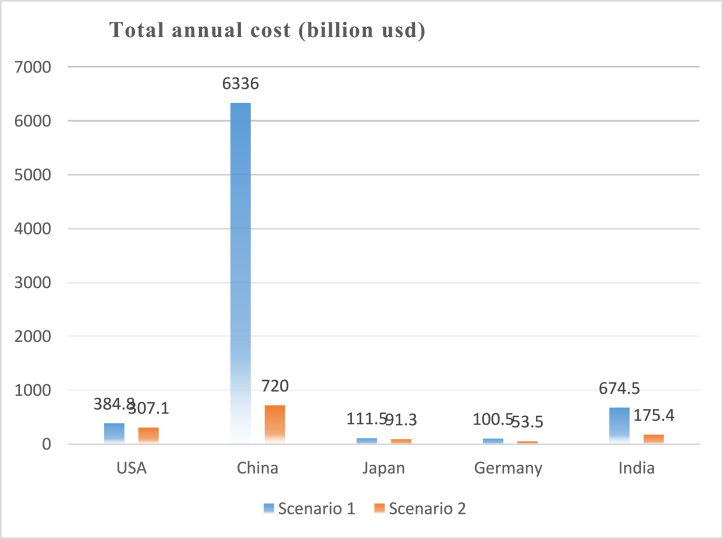
Total annual cost in both scenarios in 2030.

**Fig. 20:**
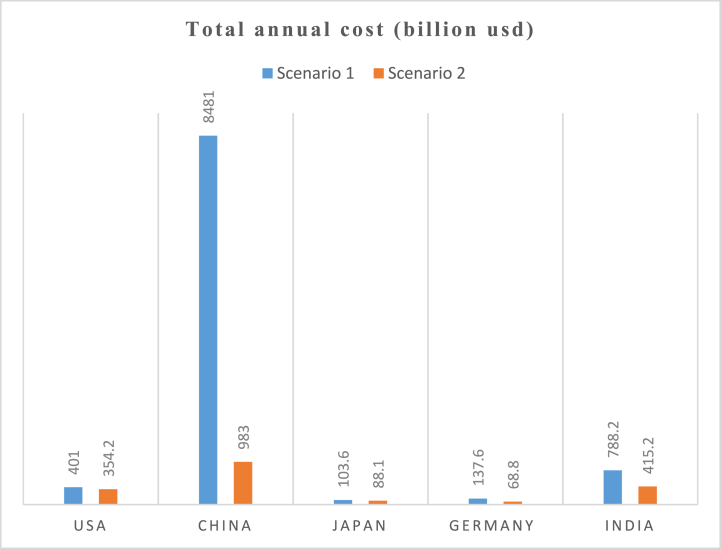
Total annual cost in both scenarios in 2050.

Fig. 19, Fig. 20 show the costs that will be incurred to achieve the CO_2_ emissions reductions in both scenarios. The total annual cost includes various factors such as investment costs, operational expenses, fuel costs, grid infrastructure upgrades, and any potential subsidies or incentives. All 5 countries will spend considerably more than the present costs in 2021. Fig. 19 shows that, compared to 367.5 billion dollars spent in 2021, the USA will spend 384 billion dollars for scenario 1 and 307.1 dollars for scenario 2. China's increase in cost will be very large for scenario 1 due to the sheer amount of CEEP that will be generated. The total annual costs for China will rise from 511 billion dollars (in 2021) to 6336 billion dollars in 2030. However, for scenario 2, the cost will only rise to 720 billion dollars. Germany's cost will rise to 103.6 billion dollars in scenario 1 and to 881 billion dollars in scenario 2, compared to 77.6 billion dollars in 2021.

The results in Fig. 20 show the same picture for total annual cost in 2050. There is also a very large cost for China in scenario 1, 8481 billion dollars while the total annual cost for scenario 2 is 983 billion dollars. India's cost will be 788.2 billion dollars for scenario 1 and 415.2 billion dollars for scenario 2, compared to 86.1 billion dollars in 2021.

It can be observed that the total annual costs in scenario 2 are less than the values for scenario 1 in all five countries. This shows that providing the storage systems that will cover all of the CEEP may not be economically sound, rather efforts can be focused on combining storage with other uses for the CEEP such as electric vehicles and hydrogen production.

Reducing the total annual costs can be achieved through various strategies such as.

• Enhancing policy frameworks and incentives and feed-in tariffs, tax credits, grants, and low-interest financing options to support renewable systems integration.
• Enhancing the grid infrastructure is crucial for integrating high shares of renewable energy. This is also applicable to electricity export schemes with neighbouring countries.
• Prioritize energy efficiency measures to reduce the electricity demand, especially during peak demand hours.

## 5 Non-Annex I countries key to Paris Agreement

According to specialists from the World Meteorological Organisation and the United Kingdom's Met Office, the past eight years have been characterised by high-temperature records. Within the next five years, we will probably experience the hottest year ever recorded. Moreover, according to their projections, the years 2023–2027 will be the warmest on record. The effects of global warming are evident and the race towards limiting global mean temperature to below 2 °C, and pursuing 1.5^o^C is on. According to the latest Intergovernmental Panel on Climate Change (IPCC), the world is not on course to meet the Paris Agreement based on the Nationally determined contributions (NDC) submitted by countries: more stringent commitments should be made towards climate mitigation actions. Based on the latest IPCC Assessment Report 6, the world must rapidly shift away from burning of fossil fuels, if the carbon budget of 510GtCO_2_ before the 2050 net-zero emission year is to be achieved. Contrary to this, future emissions from the existing, and planned fossil infrastructure is estimated to be about 850GtCO_2_. Additionally, the AR6 stated that emissions should peak by 2025 and net-zero emissions by 2050, for the world to be on the path of a 1.5 °C future. The role of the Non-Annex I countries (developing countries) has become even more critical to the task of climate change mitigation. The climate Action Tracker [38] ranks most of the Non-Annex I countries as ‘Highly Insufficient’ according to their climate targets, policy enforcement, and Climate finance. The Non-Annex I countries are legally expected to merely report emissions and are not legally bound to reduce greenhouse gas (GHG) emissions. Their roles have become critical and central in the unprecedented climate action requirements on current climate events. Six Non-Annex I countries contribute about 43% of world global emissions, and this study has highlighted the renewable energy pathway for the power sector of two of those (China, and India). 47% of the CO_2_ emission from the Non-Annex I countries results from the combustion of fossil fuels, therefore a shift towards renewable energy generation in these countries is key in realizing the Paris Agreement. Brazil, and Indonesia which make up the other top-emitting developing countries have set net-zero emission targets for 2050 and 2060 respectively. The case of Iran, which is one the last top-five emission developing nations, is quite concerning, as no commitment towards a net-zero target has been made.

The importance of proactive and ambitious policies to resolve environmental challenges is a key lesson that developing nations like Iran can learn from China regarding climate action. China has demonstrated that strong government leadership, bolstered by comprehensive strategies and objectives, can propel significant climate change mitigation progress. China's experience demonstrates the importance of establishing defined objectives and implementing policies that drive emission reductions like the development of cleaner fuels like hydrogen and strict environmental regulations. In addition, China has acknowledged the importance of international cooperation and is actively involved in global climate initiatives. By participating in international agreements such as the Paris Agreement and improving its NDC targets, China has demonstrated its commitment to addressing climate change on a global scale through collaborative efforts. This functions as a valuable lesson for developing nations such as Iran, Brazil, and Indonesia highlighting the significance of collective action in addressing climate-related issues. Furthermore, since land use and land-use change and forestry (LULUCF) constitute a significant part of emissions from the Non-Annexe I countries, sustainable land management should be key in policies among these countries. For example, Indonesia, which has significant emissions from LULUCF should create policies to utilize its forestry as a carbon sink.

## 6 Discussion and conclusion

This study has presented a comprehensive assessment of the decarbonization potential of global electricity generation by examining the transition to a high share of renewable energy in five of the world's largest economic nations and biggest pollutants: the United States, China, Japan, Germany, and India. Through the utilization of the simulation software EnergyPLAN, this study has provided valuable insights into the feasibility and implications of achieving a sustainable energy system by 2030 and 2050.

The analysis of two decarbonization scenarios has demonstrated the crucial function renewable energy may play in reducing CO_2_ emissions and advancing towards a low-carbon future. The disparity in outcomes for each nation, both in terms of the decrease in CO_2_ emissions and the overall annual cost, sheds light on the specific obstacles and opportunities that each nation faces on its path towards decarbonization. This comparative analysis is a crucial instrument for policymakers, energy planners, and interested parties in the formulation of effective energy policies.

The findings of this study reinforce the role of renewable energy and other supported policies in the goal of emission reductions in the power sector. Even though each nation faces its own unique set of obstacles, it is abundantly evident that a substantial proportion of renewable energy is essential for mitigating climate change and ensuring a sustainable future. The transition to renewable energy sources not only contributes to the reduction of greenhouse gas emissions, but also yields several additional benefits, such as increased energy security, employment creation, and enhanced air quality.

It's important to keep in mind that getting a large amount of renewable energy requires a comprehensive plan that combines policy changes, technology advances, and stakeholder participation. The establishment of comprehensive renewable energy policies, including financial incentives, regulatory frameworks, and supporting infrastructure, should be a top priority for governments. Knowledge sharing, technology transfer, and financial support for renewable energy projects can all benefit from public-private partnerships and international cooperation.

This study provides the groundwork for future decarbonization and renewable energy research and policymaking initiatives. As the need to address climate change intensifies, future studies can delve deeper into the specific challenges and opportunities encountered by individual nations and investigate novel solutions to overcome barriers to the implementation of renewable energy. In addition, advancements in simulation software and data accessibility will enhance the precision and accuracy of future assessments, enabling more nuanced and comprehensive analyses. The decarbonization of the world's electricity generation is, in conclusion, a critical challenge that requires immediate and collective action. Future study can include carbon capture and storage in the technical analysis.

## Ethical approval

Not Applicable.

## Consent to participate

Not Applicable.

## Consent to publish

The signed Consent to Publish permits the Publisher of the Author to publish the Work.

## Data availability statement

Data will be made available on request.

## CRediT authorship contribution statement

**Sandra Chukwudumebi Obiora:** Writing – original draft, Writing – review & editing, Data Curation, Conceptualization, Formal Analysis. **Olusola Bamisile:** Supervision. **Yihua Hu:** Methodology, Investigation. **Dilber Uzun Ozsahin:** Formal analysis, Writing – review & editing. **Humphrey Adun:** Writing – original draft, Formal analysis, Data curation, Conceptualization.

## Declaration of competing interest

The authors declare that there is no conflict of interest.
