# Supplementary material for: Assessing the decarbonization of electricity generation in major emitting countries by 2030 and 2050: Transition to a high share renewable energy mix
Source: Heliyon. 2024 Apr 7;10(8):e28770. doi: 10.1016/j.heliyon.2024.e28770 (PMC11031758; doi:10.1016/j.heliyon.2024.e28770)
Supplement: Multimedia component 1 [file mmc1.docx]

**Assessing the Decarbonization of Electricity Generation in Major Emitting Countries by 2030 and 2050: Transition to a High Share Renewable Energy Mix**

 Sandra Chukwudumebi Obiora^1,2+^, Olusola Bamisile^3^, Yihua Hu^4^, Dilber Uzun Ozsahin^5,6,7^, Humphrey Adun^7*+^

^1^School of Management and Economics, University of Electronic Science and Technology of China, Chengdu 611731, China.

^2^Leeds Business School, Leeds Beckett University, Leeds LS1 3HE, United Kingdom.

^3^ Electrical Engineering Department, College of Nuclear Science and Automation Engineering, Chengdu University of Technology, Sichuan P.R., Chengdu 611731, China.

^4^Electrical Engineering, Kings College London, London SE1 8WA, United Kingdom.

^5^Department of Medical Diagnostic Imaging, College of Health Science, University of Sharjah, Sharjah 27272, United Arab Emirates

^6^Research Institute for Medical and Health Sciences, University of Sharjah, Sharjah 27272, United Arab Emirates

^7^Operational Research Centre in Healthcare, Near East University, TRNC Mersin 10, Nicosia 99138, Turkey

*Corresponding author: [humphreyadun@gmail.com](mailto:humphreyadun@gmail.com)

**+ Authors can be regarded as co-first authors due to equal contribution to the work**

**EnergyPLAN Model**

The EnergyPlan model's hourly operation (8766-time steps, or the number of hours in a leap year), along with the hourly, daily, and seasonal variations in electricity and heat needs, make it appropriate for analyzing the impact of intermittent RES on the system. Every hour of the year, it aims to strike a balance between supply and demand. Distribution files, which were uploaded to the model as text files with 8784 numerical values produced outside, are a crucial input element that determines a system's hourly behaviour. Electricity demand curves (hourly network load curves), heat demand curves based on ambient temperatures, wind power curves based on wind speed, solar/PV curves based on solar radiation, and many more are typical distribution files that are employed. The model offers a choice to say whether the system being studied is connected to its surroundings. As a result, it may be used in connected mode by specifying an interconnection capacity, or in island mode, which is without connections. In the latter scenario, if technical or market economic conditions call for it, the system has the choice to import or export power at any given hour. EnergyPLAN is an input-output deterministic simulation model, but throughout the scenario's execution, various optimizations take place that prioritize renewable energy sources. The program allows users to select between two distinct simulation approaches:

1. Technical Simulation: focuses primarily on the balance between supply and demand, disregarding prices and expenses.
2. Market Simulation: based on short-term marginal costs, prices for customers are kept as low as possible.

**Input values for reference year simulation**

**Table S.1:** Input values for power systems in USA (2021)

|  | **TWh/ year** | **MW** | **Efficiency/ Share (%)** | **Capacity factor** | **GWh** | **Reference** |
| --- | --- | --- | --- | --- | --- | --- |
| **Fuel distribution** | | | | | | |
| Coal | 978 |  | 0.3559 |  |  |  |
| Oil | 20.2 |  | 0.0074 |  |  |  |
| Natural gas | 1694 |  | 0.6165 |  |  |  |
| Biomass | 55.48 |  | 0.0202 |  |  |  |
| **Variable renewable energy** | | | | | | |
| Onshore wind generation | 341.67 |  |  |  |  |  |
| Onshore wind capacity |  | 132696 |  | 0.2939 |  |  |
| PV generation | 165 |  |  |  |  |  |
| PV capacity |  | 93710 |  | 0.2010 |  |  |
| Offshore wind generation | 0.147 |  |  |  |  |  |
| Offshore wind capacity |  | 42 |  | 0.4001 |  |  |
| River hydro generation |  |  |  |  |  |  |
| River hydro capacity |  |  |  |  |  |  |
| CSP solar generation | 4.120 |  |  |  |  |  |
| CSP solar capacity |  | 1496 |  | 0.3144 |  |  |
| **Central power production** | | | | | | |
| Nuclear generation | 778.19 |  |  |  |  |  |
| Nuclear capacity |  | 94,718 | 35 | 0.9379 |  |  |
| Geothermal generation | 15.546 |  |  |  |  |  |
| Geothermal capacity |  | 2587 | 15 | 0.6860 |  |  |
| Dammed hydro generation | 260 |  |  |  |  |  |
| Dammed hydro power |  | 101943 | 90 | 0.2911 |  |  |
| Storage for dammed hydro |  |  |  |  |  |  |
| **Balancing and storage** | | | | | | |
| Pumped hydro storage |  | 21912 |  |  |  |  |
| PHS storage capacity |  |  |  |  | 550 |  |

**Table S.2:** Input values for power systems in China (2021)

|  | **TWh/ year** | **MW** | **Efficiency/ Share (%)** | **Capacity factor** | **GWh** | **Reference** |
| --- | --- | --- | --- | --- | --- | --- |
| **Fuel distribution** | | | | | | |
| Coal | 5339 |  | 0.9471 |  |  |  |
| Oil | 12.25 |  | 0.0022 |  |  |  |
| Natural gas | 273 |  | 0.0484 |  |  |  |
| Biomass | 13.2 |  | 0.0023 |  |  |  |
| **Variable renewable energy** | | | | | | |
| Onshore wind generation | 594.5 |  |  |  |  |  |
| Onshore wind capacity |  | 301682 |  | 0.2250 |  |  |
| PV generation | 327 |  |  |  |  |  |
| PV capacity |  | 306400 |  | 0.1218 |  |  |
| Offshore wind generation | 61.1 |  |  |  |  |  |
| Offshore wind capacity |  | 26798 |  | 0.2603 |  |  |
| River hydro generation | 1158 |  |  |  |  |  |
| River hydro capacity |  | 335000 |  | 0.3946 |  |  |
| CSP solar generation | 2.140 |  |  |  |  |  |
| CSP solar capacity |  | 570 |  | 0.4286 |  |  |
| **Central power production** | | | | | | |
| Nuclear generation | 407.5 |  |  |  |  |  |
| Nuclear capacity |  | 53260 | 35 |  |  |  |
| Geothermal generation | 0.1147 |  |  |  |  |  |
| Geothermal capacity |  | 26 | 15 |  |  |  |
| Dammed hydro generation | 196.97 |  |  |  |  |  |
| Dammed hydro power |  | 55920 | 90 |  |  |  |
| Storage for dammed hydro |  |  |  |  |  |  |
|  |  |  |  |  |  |  |
| **Balancing and storage** | | | | | | |
| Pumped hydro storage |  | 36000 |  |  |  |  |
| PHS storage capacity |  |  |  |  |  |  |

**Table S.3:** Input values for power systems in Japan (2021)

|  | **TWh/ year** | **MW** | **Efficiency/ Share (%)** | **Capacity factor** | **GWh** | **Reference** |
| --- | --- | --- | --- | --- | --- | --- |
| **Fuel distribution** | | | | | | |
| Coal | 302 |  | 0.4307 |  |  |  |
| Oil | 31.32 |  | 0.0447 |  |  |  |
| Natural gas | 326 |  | 0.4650 |  |  |  |
| Biomass | 41.809 |  | 0.0596 |  |  |  |
| **Variable renewable energy** | | | | | | |
| Onshore wind generation | 8.47 |  |  |  |  |  |
| Onshore wind capacity |  | 4470 |  | 0.2163 |  |  |
| PV generation | 86 |  |  |  |  |  |
| PV capacity |  | 74190 |  | 0.1323 |  |  |
| Offshore wind generation | 0.26 |  |  |  |  |  |
| Offshore wind capacity |  | 136 |  | 0.2182 |  |  |
| River hydro generation | 78 |  |  |  |  |  |
| River hydro capacity |  | 50020 |  | 0.1780 |  |  |
| CSP solar generation | 0 |  |  |  |  |  |
| CSP solar capacity |  | 0 |  |  |  |  |
| **Central power production** | | | | | | |
| Nuclear generation | 63.8 |  |  |  |  |  |
| Nuclear capacity |  | 16321 | 35 |  |  |  |
| Geothermal generation | 2.416 |  |  |  |  |  |
| Geothermal capacity |  | 525 | 15 |  |  |  |
| Dammed hydro generation |  |  |  |  |  |  |
| Dammed hydro power |  |  |  |  |  |  |
| Storage for dammed hydro |  |  |  |  |  |  |
| **Balancing and storage** | | | | | | |
| Pumped hydro storage |  | 27470 |  |  |  |  |
| PHS storage capacity |  |  |  |  |  |  |

**Table S.4:** Input values for power systems in Germany (2021)

|  | **TWh/ year** | **MW** | **Efficiency/ Share (%)** | **Capacity factor** | **GWh** | **Reference** |
| --- | --- | --- | --- | --- | --- | --- |
| **Fuel distribution** | | | | | | |
| Coal | 170.95 |  | 0.5334 |  |  |  |
| Oil | 20.78 |  | 0.0648 |  |  |  |
| Natural gas | 85.78 |  | 0.2676 |  |  |  |
| Biomass | 43 |  | 0.1342 |  |  |  |
| **Variable renewable energy** | | | | | | |
| Onshore wind generation | 87.7 |  |  |  |  |  |
| Onshore wind capacity |  | 56270 |  | 0.1779 |  |  |
| PV generation | 48.4 |  |  |  |  |  |
| PV capacity |  | 58980 |  | 0.0937 |  |  |
| Offshore wind generation | 24 |  |  |  |  |  |
| Offshore wind capacity |  | 7770 |  | 0.3526 |  |  |
| River hydro generation | 17.8 |  |  |  |  |  |
| River hydro capacity |  | 9729 |  | 0.2089 |  |  |
| CSP solar generation | 0 |  |  |  |  |  |
| CSP solar capacity |  | 0 |  |  |  |  |
| **Central power production** | | | | | | |
| Nuclear generation | 54.7 |  |  |  |  |  |
| Nuclear capacity |  | 4,055 | 35 |  |  |  |
| Geothermal generation | 0.2 |  |  |  |  |  |
| Geothermal capacity |  | 40 | 15 |  |  |  |
| Dammed hydro generation | 1.2 |  |  |  |  |  |
| Dammed hydro power |  | 924 | 90 |  |  |  |
| Storage for dammed hydro |  |  |  |  |  |  |
| **Balancing and storage** | | | | | | |
| Pumped hydro storage |  | 9800 |  |  |  |  |
| PHS storage capacity |  |  |  |  |  |  |

**Table S.5:** Input values for power systems in India (2021)

|  | **TWh/ year** | **MW** | **Efficiency/ Share (%)** | **Capacity factor** | **GWh** | **Reference** |
| --- | --- | --- | --- | --- | --- | --- |
| **Fuel distribution** | | | | | | |
| Coal | 1271 |  | 0.9441 |  |  |  |
| Oil | 2.31 |  | 0.0017 |  |  |  |
| Natural gas | 64 |  | 0.0475 |  |  |  |
| Biomass | 9.014 |  | 0.0067 |  |  |  |
| **Variable renewable energy** | | | | | | |
| Onshore wind generation | 58.13 |  |  |  |  |  |
| Onshore wind capacity |  | 39526 |  | 0.1679 |  |  |
| PV generation | 51.25 |  |  |  |  |  |
| PV capacity |  | 43607 |  | 0.1342 |  |  |
| Offshore wind generation | 0 |  |  |  |  |  |
| Offshore wind capacity |  | 0 |  | 0 |  |  |
| River hydro generation | 149.78 |  |  |  |  |  |
| River hydro capacity |  | 46337.22 |  | 0.3690 |  |  |
| CSP solar generation | 0.522 |  |  |  |  |  |
| CSP solar capacity |  | 343 |  | 0.1737 |  |  |
| **Central power production** | | | | | | |
| Nuclear generation | 43.92 |  |  |  |  |  |
| Nuclear capacity |  | 6,795 | 35 |  |  |  |
| Geothermal generation | 0 |  |  |  |  |  |
| Geothermal capacity |  | 0 | 15 |  |  |  |
| Dammed hydro generation |  |  |  |  |  |  |
| Dammed hydro power |  |  | 90 |  |  |  |
| Storage for dammed hydro |  |  |  |  |  |  |
| **Balancing and storage** | | | | | | |
| Pumped hydro storage |  | 4746 |  |  |  |  |
| PHS storage capacity |  |  |  |  |  |  |

**Input values for financial data for all countries**

**Table S.6:** Input financial values for 2021

|  | **Million USD per unit** | **Years** | **O&M (% of investment cost)** | **USD/GJ** | **USD/MWhe** |
| --- | --- | --- | --- | --- | --- |
| **Heat and Electricity** | | | | | |
| Large power plants | 1.36 | 25 | 2.78 |  |  |
| Nuclear | 4.12 | 60 | 1.9 |  |  |
| Interconnection | 1.2 | 40 | 1 |  |  |
| Charge el1 storage | 0.6 | 50 | 1.5 |  |  |
| Discharge el1 storage | 0.6 | 50 | 1.5 |  |  |
| El1 storage cap | 7.5 | 50 | 1.5 |  |  |
| Charge el2 storage | 0 | 0 | 0 |  |  |
| Discharge el2 storage | 0 | 0 | 0 |  |  |
| El2 storage cap | 0 | 0 | 0 |  |  |
| **Renewable Energy** | | | | | |
| Wind | 0.77 | 30 | 1.64 |  |  |
| Wind offshore | 1.93 | 30 | 1.87 |  |  |
| Photo Voltaic | 0.63 | 40 | 1.47 |  |  |
| CSP | 5.98 | 25 | 8.21 |  |  |
| River hydro | 3.3 | 50 | 2 |  |  |
| Hydro Power | 3.3 | 50 | 2 |  |  |
| Hydro Storage | 7.5 | 50 | 1.5 |  |  |
| Hydro Pump | 0.6 | 50 | 1.5 |  |  |
| Geothermal Electric | 4.03 | 20 | 3.48 |  |  |
| **Fuel** | | | | | |
| Coal |  |  |  | 2.9 |  |
| Fuel Oil |  |  |  | 10.4 |  |
| Natural gas |  |  |  | 6.6 |  |
| Biomass |  |  |  | 6.6 |  |
| Nuclear Uranium |  |  |  | 1.5 |  |
| **Variable O & M Cost** | | | | | |
| Hydro Power |  |  |  |  | 1.19 |
| Condensing |  |  |  |  | 2.92 |
| Geothermal |  |  |  |  | 15 |
| Pump |  |  |  |  | 1.19 |
| Turbine |  |  |  |  | 1.19 |

**Table S.7:** Input financial values for 2030

|  | **Million USD per unit** | **Years** | **O&M (% of investment cost)** | **USD/GJ** | **USD/MWhe** |
| --- | --- | --- | --- | --- | --- |
| **Heat and Electricity** | | | | | |
| Large power plants | 1.487 | 25 | 1.64 |  |  |
| Nuclear | 4.12 | 60 | 1.9 |  |  |
| Interconnection | 1.2 | 40 | 1 |  |  |
| Charge el1 storage | 0.6 | 50 | 1.5 |  |  |
| Discharge el1 storage | 0.6 | 50 | 1.5 |  |  |
| El1 storage cap | 7.5 | 50 | 1.5 |  |  |
| Charge el2 storage | 0.6 | 50 | 1.5 |  |  |
| Discharge el2 storage | 0.6 | 50 | 1.5 |  |  |
| El2 storage cap | 7.5 | 50 | 1.5 |  |  |
| **Renewable Energy** | | | | | |
| Wind | 1.08 | 30 | 1.25 |  |  |
| Wind offshore | 2.03 | 30 | 1.87 |  |  |
| Photo Voltaic | 0.715 | 40 | 1.45 |  |  |
| CSP | 5.98 | 25 | 8.21 |  |  |
| River hydro | 3.3 | 50 | 2 |  |  |
| Hydro Power | 3.3 | 50 | 2 |  |  |
| Hydro Storage | 7.5 | 50 | 1.5 |  |  |
| Hydro Pump | 0.6 | 50 | 1.5 |  |  |
| Geothermal Electric | 4.03 | 20 | 3.48 |  |  |
| **Fuel** | | | | | |
| Coal |  |  |  | 2.9 |  |
| Fuel Oil |  |  |  | 10.4 |  |
| Natural gas |  |  |  | 6.6 |  |
| Biomass |  |  |  | 6.6 |  |
| Nuclear Uranium |  |  |  | 1.5 |  |
| **Variable O & M Cost** | | | | | |
| Hydro Power |  |  |  |  | 1.19 |
| Condensing |  |  |  |  | 2.92 |
| Geothermal |  |  |  |  | 15 |
| Pump |  |  |  |  | 1.19 |
| Turbine |  |  |  |  | 1.19 |

**Table S.8:** Input financial values for 2050

|  | **Million USD per unit** | **Years** | **O&M (% of investment cost)** | **USD/GJ** | **USD/MWhe** |
| --- | --- | --- | --- | --- | --- |
| **Heat and Electricity** | | | | | |
| Large power plants | 0.53 | 25 | 3.5 |  |  |
| Nuclear | 4.12 | 60 | 1.9 |  |  |
| Interconnection | 1.2 | 40 | 1 |  |  |
| Charge el1 storage | 0.6 | 50 | 1.5 |  |  |
| Discharge el1 storage | 0.6 | 50 | 1.5 |  |  |
| El1 storage cap | 7.5 | 50 | 1.5 |  |  |
| Charge el2 storage | 0.6 | 50 | 1.5 |  |  |
| Discharge el2 storage | 0.6 | 50 | 1.5 |  |  |
| El2 storage cap | 7.5 | 50 | 1.5 |  |  |
| **Renewable Energy** | | | | | |
| Wind | 1.03 | 30 | 1.67 |  |  |
| Wind offshore | 1.9 | 30 | 2.51 |  |  |
| Photo Voltaic | 0.6 | 40 | 1.5 |  |  |
| CSP | 5.98 | 25 | 8.21 |  |  |
| River hydro | 3.3 | 50 | 2 |  |  |
| Hydro Power | 3.3 | 50 | 2 |  |  |
| Hydro Storage | 7.5 | 50 | 1.5 |  |  |
| Hydro Pump | 0.6 | 50 | 1.5 |  |  |
| Geothermal Electric | 4.03 | 20 | 3.48 |  |  |
| **Fuel** | | | | | |
| Coal |  |  |  | 3 |  |
| Fuel Oil |  |  |  | 12.5 |  |
| Natural gas |  |  |  | 7.8 |  |
| Biomass |  |  |  | 6.9 |  |
| Nuclear Uranium |  |  |  | 1.75 |  |
| **Variable O & M Cost** | | | | | |
| Hydro Power |  |  |  |  | 1.19 |
| Condensing |  |  |  |  | 2.92 |
| Geothermal |  |  |  |  | 15 |
| Pump |  |  |  |  | 1.19 |
| Turbine |  |  |  |  | 1.19 |

**Figure** S1**:** Hourly electricity profile for energy demand, nuclear energy, and hydroelectricity in 2030 for China

**Figure** S2**:** Hourly electricity profile for wind energy, solar energy, CEEP, and fossil fuel power plants in 2030 for China

**Figure S3:** Hourly electricity profile for energy demand, nuclear energy, and hydroelectricity in 2050 for China

**Figure** S4**:** Hourly electricity profile for wind energy, solar energy, CEEP, and fossil fuel power plants in 2030 for China

**Figure** S5**:** Hourly electricity profile for energy demand, nuclear energy, and hydroelectricity in 2030 for Japan

**Figure** S6**:** Hourly electricity profile for wind energy, solar energy, CEEP, and fossil fuel power plants in 2030 for Japan

**Figure S7:** Hourly electricity profile for energy demand, nuclear energy, and hydroelectricity in 2050 for Japan

**Figure** S8**:** Hourly electricity profile for wind energy, solar energy, CEEP, and fossil fuel power plants in 2050 for Japan

**Figure** S9**:** Hourly electricity profile for energy demand, nuclear energy, and hydroelectricity in 2030 for Germany

**Figure** S10**:** Hourly electricity profile for wind energy, solar energy, CEEP, and fossil fuel power plants in 2030 for Germany

**Figure** S11**:** Hourly electricity profile for energy demand, nuclear energy, and hydroelectricity in 2050 for Germany

1. **Figure** S12**:** Hourly electricity profile for wind energy, solar energy, CEEP, and fossil fuel power plants in 2050 for Germany

**Figure** S13**:** Hourly electricity profile for energy demand, nuclear energy, and hydroelectricity in 2030 for India

**Figure** S14**:** Hourly electricity profile for wind energy, solar energy, CEEP, and fossil fuel power plants in 2030 for India

**Figure** S15**:** Hourly electricity profile for energy demand, nuclear energy, and hydroelectricity in 2050 for India

**Figure S16:** Hourly electricity profile for wind energy, solar energy, CEEP, and fossil fuel power plants in 2050 for India
